# Hinge action versus grip in translocation by RNA polymerase

**DOI:** 10.1080/21541264.2017.1330179

**Published:** 2017-08-30

**Authors:** Yuri A. Nedialkov, Kristopher Opron, Hailey L. Caudill, Fadi Assaf, Amanda J. Anderson, Robert I. Cukier, Guowei Wei, Zachary F. Burton

**Affiliations:** a Department of Biochemistry and Molecular Biology, Michigan State University, E. Lansing, MI, USA; b Department of Microbiology, The Ohio State University, Columbus, OH, USA; c Department of Mathematics, Michigan State University, E. Lansing, MI, USA; d Bioinformatics Core, North Campus Research Complex (NCRC), Ann Arbor, MI, USA; e Department of Chemistry, Michigan State University, E. Lansing, MI, USA

**Keywords:** Bridge helix and trigger loop hinges, hyper-translocation in termination, molecular dynamics simulation, RNA polymerase translocation, RNA threading through the exit channel, ternary elongation complex, trigger loop dynamics

## Abstract

Based on molecular dynamics simulations and functional studies, a conformational mechanism is posited for forward translocation by RNA polymerase (RNAP). In a simulation of a ternary elongation complex, the clamp and downstream cleft were observed to close. Hinges within the bridge helix and trigger loop supported generation of translocation force against the RNA–DNA hybrid resulting in opening of the furthest upstream i−8 RNA–DNA bp, establishing conditions for RNAP sliding. The β flap tip helix and the most N-terminal β′ Zn finger engage the RNA, indicating a path of RNA threading out of the exit channel. Because the β flap tip connects to the RNAP active site through the β subunit double-Ψ–β-barrel and the associated sandwich barrel hybrid motif (also called the flap domain), the RNAP active site is coupled to the RNA exit channel and to the translocation of RNA–DNA. Using an exonuclease III assay to monitor translocation of RNAP elongation complexes, we show that K^+^ and Mg^2+^ and also an RNA 3′-OH or a 3′-H_2_ affect RNAP sliding. Because RNAP grip to template suggests a sticky translocation mechanism, and because grip is enhanced by increasing K^+^ and Mg^2+^concentration, biochemical assays are consistent with a conformational change that drives forward translocation as observed in simulations. Mutational analysis of the bridge helix indicates that 778-GARKGL-783 (*Escherichia coli* numbering) is a homeostatic hinge that undergoes multiple bends to compensate for complex conformational dynamics during phosphodiester bond formation and translocation.

## Introduction

Multi-subunit RNA polymerases (RNAPs) are of the two double-Ψ-β-barrel (DPBB) type.^1-3^ The active site is between the β and β′ subunit DPBBs and also the β′ bridge helix and trigger loop.^4-6^ The DPBBs can be thought of as a compact core of the RNAP thermal translocation ratchet, both helping to drive translocation and also restraining translocation to single steps. The trigger loop is a mobile element that can close and tighten to support phosphodiester bond synthesis or open to support translocation. The bridge helix and the closed trigger loop interact to form a fairly tight three helix bundle associated with the chemical step in RNA synthesis. The active site of RNAP is buried deep within the clamp and cleft, and dynamics of the “crab claw” domains can increase or restrain the grip of RNAP for the RNA–DNA hybrid and for downstream DNA.

RNAP translocation has been described as an unrestrained thermal ratchet with rapid and reversible transitions between the pre-translocated and post-translocated registers.^7-16^ In crystal structures and using exonuclease mapping of TEC boundaries, however, RNAP appears to mostly reside in the post-translocated register, indicating that at many template positions the rate of forward translocation is fast compared to rates of reverse translocation.^5,6^ For these and other reasons, RNAP has been considered to translocate via conformational changes.^17,18^ The models are not necessarily mutually exclusive, and the preferred model may depend on the relative grip of RNAP for RNA–DNA, which might be tuned by altering buffer conditions. In this paper, we support a conformational model for forward translocation based on molecular dynamics simulations complemented by functional studies. Notably, increases in K^+^ (40 versus 120 mM) and Mg^2+^ (0 versus 5 mM) concentrations are found to enhance RNAP grip for RNA–DNA to modulate translocation.

All atom molecular dynamics simulations can potentially give insight into protein conformations that are not fully represented by available x-ray crystal structures. Many structures of RNAP ternary elongation complexes (TECs) are available, but only a small number are reported with a closed trigger loop conformation, which is thought to be the catalytic form. For *Thermus thermophilus* (Tt) RNAP, the PDB 2O5J structure appears to have a closed trigger loop.^4^ For *Saccharomyces cerevisiae* RNAP II, the PDB 2E2H structure appears to represent a nearly closed, catalytic conformation, but the trigger loop in 2E2H is not as fully closed or as helical as in 2O5J.^6^ In the present simulation, we started with the Tt RNAP PDB 2O5J TEC structure, with a closed trigger loop, but, as the simulation progressed, the trigger loop tightened relative to the initial conformation. The simulation posits a simple but unexpected conformational model for RNAP translocation that appears consistent with functional studies.

Two major bend points have been identified on the long bridge α-helix.^19-22^ The N-terminal hinge GARKG appears to be the primary bend point. A C-terminal hinge GY has also been identified. Bending or thermal vibration of the bridge helix is posited to generate translocation force on the RNA–DNA hybrid, and, therefore, bridge helix dynamics in translocation could be supported by hinge bending. When the trigger loop is closed, extended trigger helices form, but each helix includes a GXP (X can be many different amino acids) hinge. In the bacterial RNAP trigger helices, hinges have the sequences GEPG and GLP. Simulations show dramatic action of the more C-terminal GLP hinge associated with forward translocation. Mutational analyses support the importance of the bridge helix and trigger loop hinges.^19-25^

To better understand translocation, the effects of monovalent and divalent ions on RNAP TEC sliding were analyzed. Also, a 3′-OH and a 3′-H_2_ RNA end can differentially affect TEC translocation. Buffer conditions and the RNA 3′-end, therefore, can be altered to tune sliding of the RNAP TEC. These assays demonstrate that RNAP has significant grip for RNA–DNA, which can be increased by increasing K^+^ and Mg^2+^concentration. K^+^ may indirectly affect grip by affecting RNA–DNA melting at the RNA 3′-end. Mg^2+^, by contrast, appears directly to enhance grip, i.e., by bridging interactions of aspartic acid with phosphates. RNAP grip for the TEC is consistent with a conformational model for TEC sliding during translocation.

## Results

### Molecular dynamics simulations

#### Bending of the trigger loop hinges in translocation

**Figure 1.**
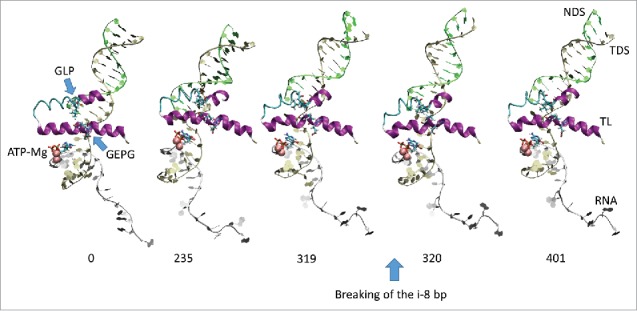
Trigger loop overtightening. Dynamics is detected in Tt RNAP trigger loop GXP hinges 1230-GEPG-1233 and 1255-GLP-1257. RNA is silver; TDS (template DNA strand) is gold; NDS (non-template DNA strand) is green. Protein is in secondary structure representation: helix is purple; β-sheets are yellow; turn is white; coil is cyan. Frame numbers 0–401 are indicated, representing ∼100 ns total simulation time.

**Figure 2.**
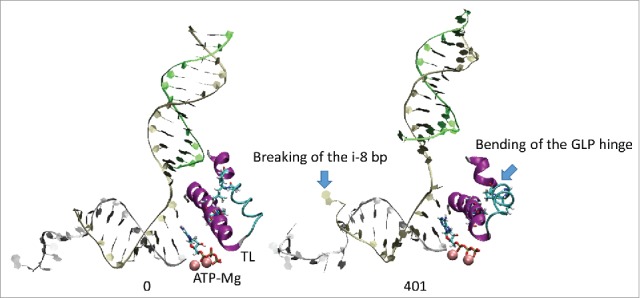
We posit that trigger loop overtightening and the associated conformational change in RNAP result in breaking of the i−8 RNA–DNA bp, which is a prerequisite for forward translocation.

**Figure 3.**
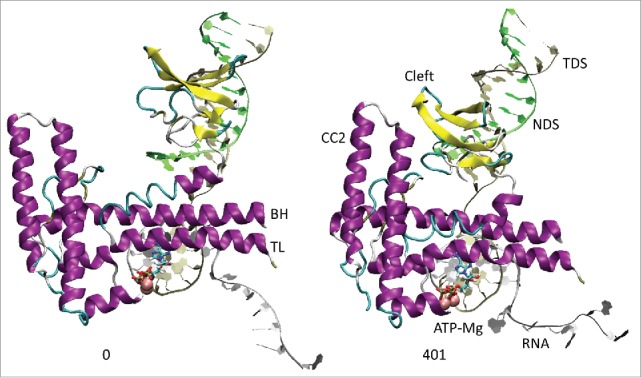
The coiled coil 2 (CC2; β′ 958–1014) and cleft (β′ 1279–1319) move into close proximity during the simulation. Multiple connecting ion pairs form (see Movie 3).

**Figure 4.**
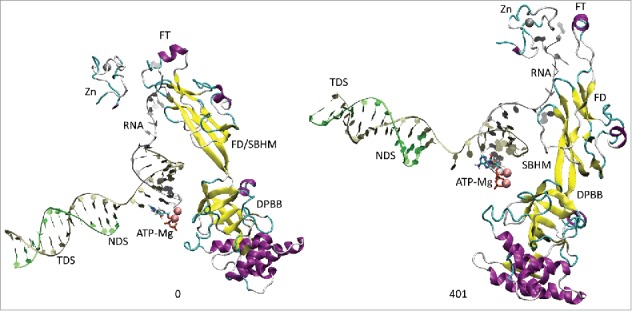
The RNA exit path is remodeled during the simulation to bring the Zn finger (β′ 54–82) and the flap tip helix (β 768–780) close to the RNA. The RNA exit channel is coupled to the active site through the β DPBB (β 670–997). FD indicates the flap domain; SBHM indicates the sandwich barrel hybrid motif (an evolutionary designation). The active site, therefore, connects to the RNA exit channel (active site→DPBB→FD (SBHM)→flap tip helix→RNA).

All atom molecular dynamics simulation was used to analyze translocation by Tt RNAP (Figs. 1–4; Supplemental Movies 1–3). The simulation shown indicates that generation of translocation force has a conformational component in which the bridge helix, the trigger loop, the clamp, the cleft and the RNA exit channel (the β flap tip helix and the most N-terminal β′ Zn finger) are involved. The trigger loop (β′ 1221–1263; Tt RNAP numbering) and the bridge helix (β′ 1066–1103) cooperate to help generate force on the RNA–DNA hybrid. Bending and dynamics at two GXP hinges (Tt RNAP 1230-GEPG-1233 and 1255-GLP-1257) within the trigger loop helical segments facilitates generation of force. In the simulation shown, hinge action is noted at both trigger loop hinges, and dramatic bending occurs at the more C-terminal trigger loop hinge 1255-GLP-1257 (Figs. 1 and 2 and Movie 1). A strong indication of translocation force is the breaking of the i−8 RNA–DNA bp between frames 319 and 320 (400 frames = 100 ns simulation) (Figs. 1 and 2). Breaking of the i−8 bp, the most upstream bp in the RNA–DNA hybrid, is necessary before forward sliding of the TEC can occur. The dramatic change of conformation of the trigger loop seen in the simulation has not been observed in x-ray crystal structures, although only a few closed trigger loop TEC structures are available. This result indicates the utility of molecular dynamics to probe energetically accessible conformational states that might otherwise not be observed. Clamp closure accompanies the conformational change, which extends throughout Tt RNAP. Fig. 3 shows the closing of the cleft, bringing CC2 (coiled coil 2; β′ 958–1014; Tt numbering) close to a set of β-sheets just C-terminal to the trigger loop (i.e., β′ 1279–1319) (see Movie 3). Fig. 4 shows the remodeling of the RNA exit channel during the simulation. In the 2O5J crystal structure, the 16-nt RNA is almost long enough to fill the RNA exit channel. In the initial simulation frame, the Zn finger (β′ 54–82) is only partially engaged, but, by the end of the simulation, the Zn finger is fully engaged with the exiting RNA (Movie 2). At the end of the simulation, the flap tip helix also engages the exiting RNA. The flap tip helix (β 768–780) is at the tip of a loop formed between β sheets 2 and 3 of the 6 sheet β subunit DPBB (β 670–997). The evolutionary designation for the RNAP flap domain is a sandwich barrel hybrid motif.^3^ We propose that a route for threading RNA during active elongation is observed in the simulation. Because the DPBB connects to the RNAP active site, and because the flap tip helix is part of a loop of the DPBB, RNA exit can be coupled to catalysis and translocation. The simulation indicates the importance of hinge action, trigger loop tightening, clamp closing, cleft closing and RNA threading in translocation.

The conformational change depicted in the simulation is also shown in three movies. Movie 1 indicates the dynamics and bending of the trigger loop. Movie 2 shows the remodeling of the RNA exit channel and the repositioning of the flap tip helix and Zn finger. Movie 3 shows the closing of the downstream cleft. This is a large conformational change that extends throughout RNAP. In the current simulations, clamp closure and RNAP grip for RNA–DNA is neither abetted nor restrained by elongation factors.

### Mutagenesis of the bridge helix N-terminal hinge

**Figure 5.**
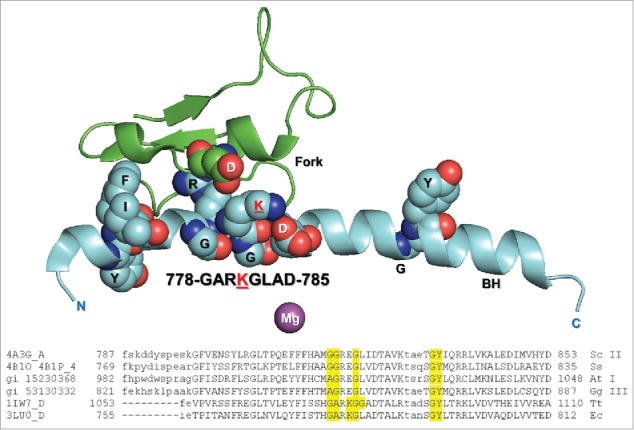
The Ec RNAP bridge helix highlighting the β′ 778-GARKGL-783 N-terminal hinge. R780 penetrates the β fork. A broad alignment of bridge helix sequences is shown. Sc II: *Saccharomyces cerevisiae* RNAP II; Ss: *Sulfolobus solfataricus* RNAP (archaeal); At I: *Arabidopsis thaliana* RNAP I; Gg III: *Gallus gallus* RNAP III; Tt: *Thermus thermophilus* RNAP; Ec: *Escherichia coli* RNAP.

To better understand *Escherichia coli* (Ec) RNAP dynamics and function, a number of substitutions were generated in and around the N-terminal bridge helix hinge, which cooperates with the two trigger loop GXP hinges. Both the GEPG and GLP trigger loop hinges are very active in the simulation shown (Movie 1), and the GLP hinge bends, apparently generating translocation force on the RNA–DNA hybrid. Fig. 5 shows the Ec RNAP β′ bridge helix and β fork, emphasizing potential bridge helix N-terminal hinge residues. The broad alignment shows that N-terminal bridge helix hinge character is conserved across species, although sequence differences indicate that hinge bending and dynamics are likely modulated differently for different RNAPs. For Ec RNAP, the bridge helix N-terminal hinge sequence is 778-GARKGL-783. The head group of R780 is enveloped by a loop of the β fork. K781 can form an ion pair with β D546 on the fork or with β′ D785 on the bridge helix. The two hinge glycines, β′ G778 and G782, are expected to be bend points.

**Figure 6.**
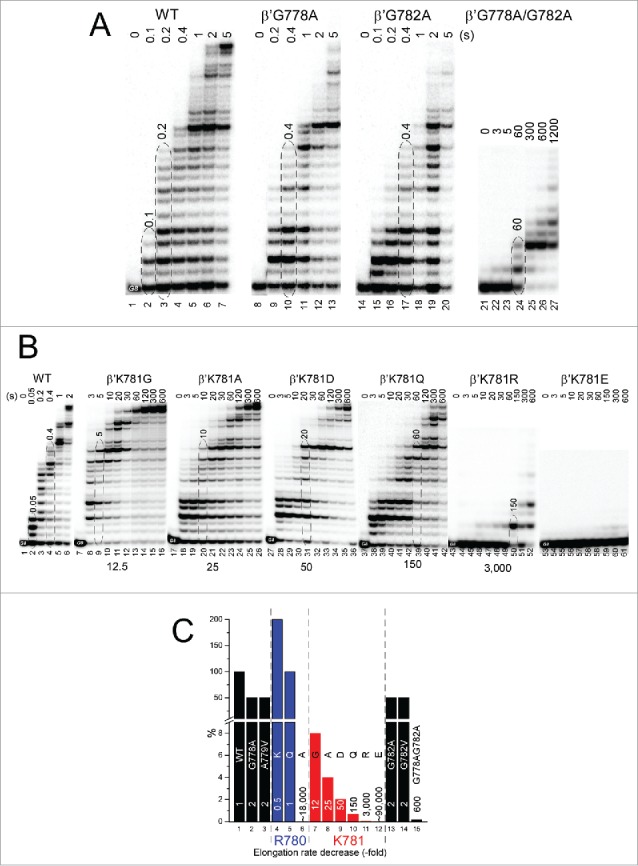
Transcriptional activities of hinge mutants. (A) Combinatorial effects of G778 and G782 hinge mutants. (B) K781 substitutions. (C) Estimated relative transcription rates (fold reduction relative to wild-type RNAP).

To gain insight into the bridge helix N-terminal hinge, transcription assays are shown (Fig. 6). TECs were assembled in vitro, and 5′-^32^P-labeled RNA was extended from the G8 position by addition of 200 μM NTPs. Fig. 6A emphasizes the strong combinatorial effects of alanine substitutions for glycines in the hinge. Single G778A or G782A substitutions are only ∼2-fold down for transcriptional activity compared to wild-type RNAP, but the G778A/G782A double substitution is ∼600-fold down for elongation. We conclude that at least one of the two glycines must be present for normal hinge activity and, for the most part, either glycine will suffice to support bending, when the other is substituted with alanine. The large synergistic effect observed with substitution of both glycines to alanine is most consistent with a complex hinge with multiple bend points and complex dynamics. Fig. 6B highlights K781G/A/D/Q/R/E substitutions (summarized in Fig. 6C). All substitutions of K781 are strongly defective for elongation. K781G is ∼12-fold down compared to wild-type RNAP. K781A is ∼25-fold down.^23^ K781D, which substitutes a negative for a positive charge, is ∼50-fold down. K781Q is ∼150-fold down. Remarkably, the K781R substitution, which might be considered a “conservative” substitution because it maintains a positive charge, is ∼3000-fold down in transcription rate. Remarkably, K781E is ∼90,000 fold slower than wild-type RNAP. The K781E substitution is of interest because in archaeal and eukaryotic RNAPs, the residue corresponding to Ec RNAP β′ K781 is glutamate, consistent with different hinge modulation in different RNAPs. The transcriptional activities of these and other hinge mutants are estimated in Fig. 6C.

**Figure 7.**
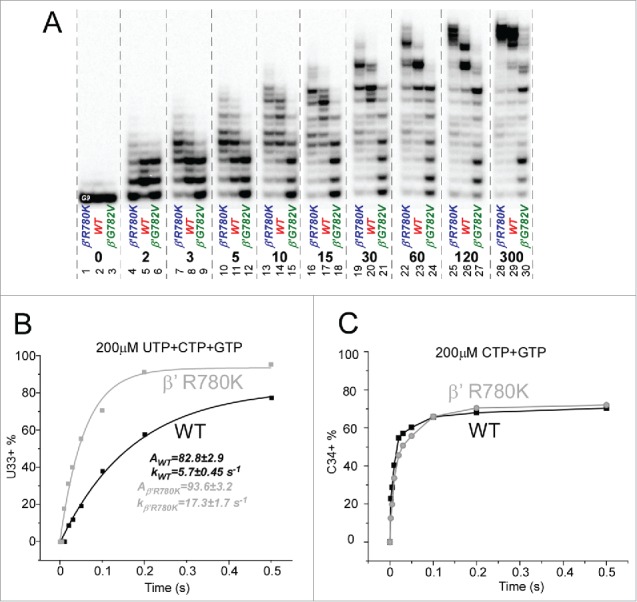
β′R780K is a fast RNAP mutant that suppresses pausing at some sites. (A) Transcription rates of RNAPs. (B) RNAP escapes from pausing at A32. (C) RNAP escapes from stalling at U33.

β′ R780 is highly conserved (Fig. 5) and surrounded by β fork residues forming multiple main chain hydrogen bonds but no close ion pairs. The arrangement resembles a ball-in-socket joint in which the arginine head group is the ball and a loop of the fork loop forms the socket (Fig. 5). Interestingly, R780K, with its more flexible side chain and more compact positive charge, is ∼2-fold faster in elongation than wild-type RNAP (Figs. 6C and 7A). R780K fails to acknowledge some pause sites recognized by wild-type RNAP (Fig. 7B). In the example shown, R780K is much faster than wild-type RNAP for the A32→U33 transition, which requires escape from the A32 pause (Fig. 7B; left panel). On the other hand, rates for the U33→C34 transition, which is not as dependent on pause escape, are indistinguishable for R780K and wild-type RNAP (Fig. 7B; right panel). R780Q substitution is not noticeably defective for transcription (Fig. 6C). By strong contrast, R780A, which cannot interact with the fork, is ∼18,000-fold reduced in transcriptional activity compared to wild-type RNAP.^23^ Probably, R780 connects the bridge helix N-terminal hinge to the fork to coordinate hinge dynamics. R780K and R780Q can partly mimic R780 function in bridge-fork coupling. Interestingly, R780K, which is rapid in elongation, has an enhanced affinity for NTP substrates (Table 1). R780Q is similar to wild type in NTP affinity.

**Table 1.** *K*_D_ and *k*_pol_ of selected hinge mutants. Error is standard error.

RNAP	WT	R780K	R780Q	K781A	G778A	G778A/G782A
*k*_pol_ (s^−1^)	65.2 ± 0.6	58.1 ± 3	26.4 ± 1.1	6.9 ± 0.2	53.7 ± 5.3	0.04 ± 0.001
*K*_D_ (µM)	64.8 ± 2.6	5.5 ± 1.3	50.1 ± 6.1	545 ± 41	51.2 ± 10.6	155 ± 25

**Figure 8.**
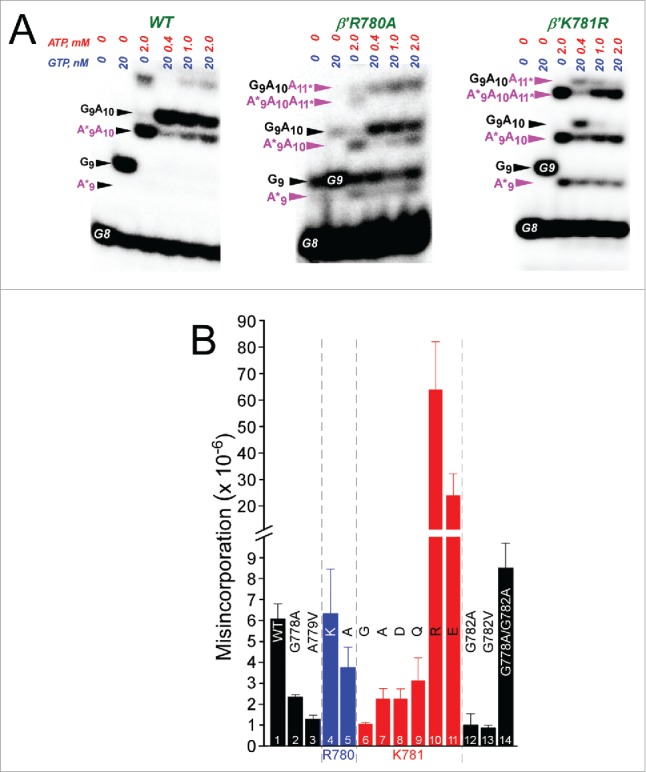
Transcriptional fidelity of hinge mutants. (A) Selected competition fidelity assays. R780A is very slow in elongation but slightly higher fidelity than wild type. K781R is very slow in elongation but error prone. (B) Quantification of competition fidelity assays. Error is standard deviation of three measurements.

N-terminal hinge substitutions also strongly affect transcriptional fidelity (Fig. 8). Here, misincorporation is judged as a competition between incorporation of the accurate GTP (encoded at position G9), at low concentration (20 nM), and an inaccurate competing ATP (A*9), at a high concentration (0.4, 1 and 2 mM).^26,27^ Fig. 8A shows some examples of competition fidelity assays. Fig. 8B shows quantification of fidelity for several hinge mutants. G778A, A779V, G782A, G782V and K781A/Q/D/G have significantly enhanced fidelity compared to wild-type RNAP. R780A, which is very slow in elongation, has somewhat higher fidelity than wild-type RNAP. Two other very slow mutants, however, K781E/R, are highly error-prone, showing that different very slow hinge mutants can demonstrate either high or very low fidelity using the competition misincorporation assay. Therefore, very slow RNAP mutants do not necessarily score as low or as high fidelity mutants, and fast or very slow rates of elongation do not appear to affect the reliability of the competition fidelity assay.

### Mapping TEC boundaries

**Figure 9.**
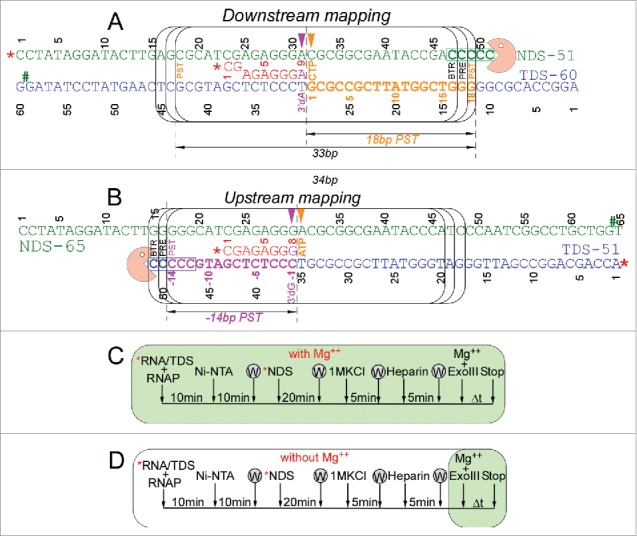
TEC assembly and exonuclease III mapping protocols. (A) Downstream RNAP TEC border mapping protocol and interpretation. (B) Upstream TEC border mapping protocol and interpretation. NDS indicates non-template DNA strand. TDS indicates template DNA strand. The RNA is red. Post-translocated (PST), pre-translocated (PRE) and backtracked (BTR) states of RNAP are indicated. # indicates the position of a thio-NMP to block exonuclease III digestion on one stand. * indicates a 5′-^32^P label. (C) Assembly of TECs in the presence of 5 mM MgCl_2_. (D) Assembly of TECs in the absence of MgCl_2_. In this case, MgCl_2_ is added with exonuclease III. W indicates washing of complexes immobilized on beads. TECs were released from beads with imidazole before assays.

Fig. 9 shows the methods used for exonuclease III mapping of TEC boundaries and assembly of RNAP TECs. The protocol for downstream TEC border mapping is shown in Fig. 9A, along with its interpretation. The protocol for upstream TEC border mapping is shown in Fig. 9B. Fig. 9C shows a method for TEC assembly in the presence of 5 mM MgCl_2_. Fig. 9D shows a method for TEC assembly in the absence of MgCl_2_. It was found that omitting MgCl_2_ during TEC assembly caused increased slippage of TECs, particularly for β′ K781Q/R/E substitutions.

**Figure 10.**
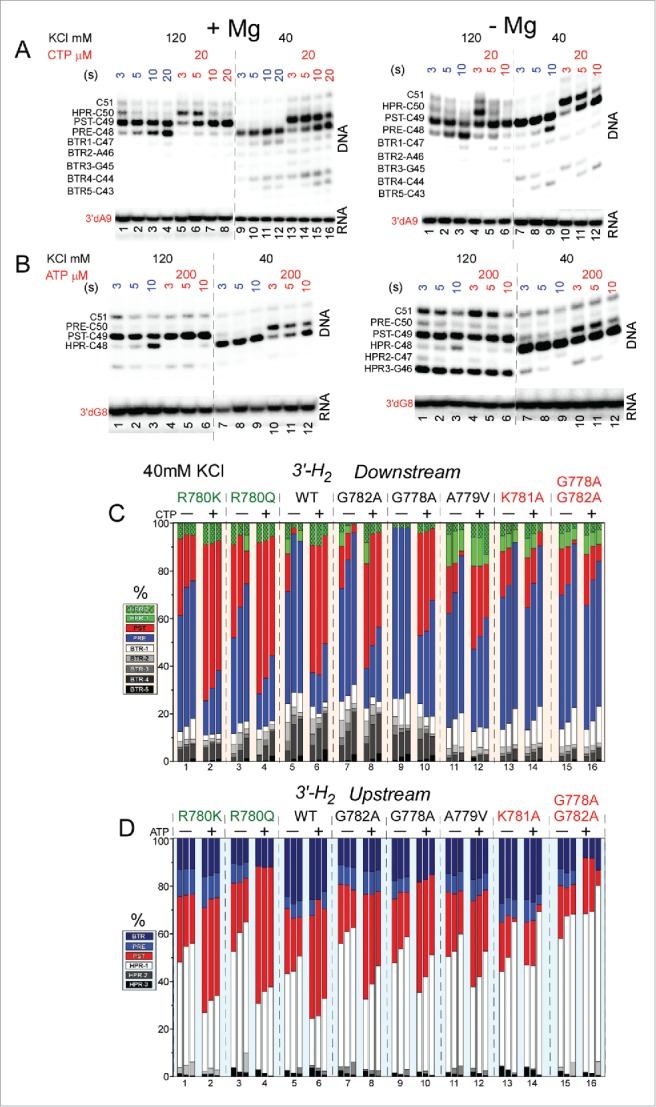
Exonuclease III mapping of TEC boundaries. (A) Mapping of the downstream wild-type RNAP TEC boundary. (Left panel) TECs were assembled in the presence of 5 mM MgCl_2_. (Right panel) TECs were assembled in the absence of 5 mM MgCl_2_, increasing TEC sliding (see Fig. 9). (B) Mapping of the upstream TEC boundary. (Left panel) TECs were assembled in the presence of 5 mM MgCl_2_. (Right panel) TECs were assembled in the absence of 5 mM MgCl_2_, increasing TEC sliding. (C) Exonuclease mapping of the downstream TEC border for selected RNAP mutants. Mapping is of TECs formed in the presence of 5 mM MgCl_2_ at 40 mM KCl. (D) Exonuclease mapping of the upstream TEC border for selected mutants. Mapping is of TECs formed in the presence of 5 mM MgCl_2_ at 40 mM KCl. Sets of 3 bars represent 3, 5 and 10 seconds treatment with exonuclease III.

Fig. 10 shows exonuclease III mapping of upstream and downstream RNAP borders. Fig. 10A and B shows mapping for wild-type RNAP. Fig. 10A shows mapping of the downstream wild-type RNAP boundary (see Fig. 9A for protocol). Fig. 10B shows mapping of the upstream wild-type RNAP boundary (see Fig. 9B). Left panels show mapping of TECs assembled in the presence of 5 mM MgCl_2_ (see Fig. 9C), and right panels show mapping of TECs generated in the absence of MgCl_2_ (see Fig. 9D). When TECs were assembled without adding MgCl_2_, Mg^2+^ was added with exonuclease III to support 3′→5′ DNA digestion. When MgCl_2_ was added only after TEC assembly, however, TECs have a tendency to slide on the DNA. Because TEC sliding is enhanced at 40 mM KCl compared to 120 mM KCl, mapping was done in 40 and 120 mM KCl buffers. For instance, backtracking (Fig. 10A) was more apparent at 40 mM KCl than at 120 mM KCl. The post-translocated TEC was stabilized at 120 mM KCl compared to 40 mM KCl (see Fig. 10A and B, left panels). Addition of an accurately loaded NTP, particularly stabilizes the post-translocated register. Hyper-translocation can lead to termination (Fig. 10B, right panel). Termination is most apparent when TECs are assembled in the absence of MgCl_2_ and KCl is at 40 mM. At 120 mM KCl, termination is inhibited. The HPR4 (HPR to indicate the hyper-translocated TEC, in this case, by four base steps) TEC is not observed, indicating that termination occurs at the HPR3→HPR4 transition.

To map RNAP excursions upstream, toward the pre-translocated and backtracked states, exonuclease III was used to attack the downstream TEC boundary on the non-template DNA strand (3′→5′) (Fig. 10A and C). For the experiment shown in Fig. 10C, all mapping was done in buffer containing 40 mM KCl. A RNA 3′-H_2_ chain terminator is present to block incorporation of CTP (the next accurately templated NTP). For the wild-type RNAP, in the absence of CTP, the pre-translocated TEC is mostly detected over the post-translocated TEC. This result is expected because during the exonuclease III digestion, the TEC is likely to return to the pre-translocated position within 3 seconds even if resting TECs are mostly post-translocated. In the presence of CTP, the post-translocated TEC is strongly stabilized over the pre-translocated TEC, as expected. In the presence of CTP, the pre-translocated TEC is observed because of the long times of exonuclease III digestion relative to reverse translocation rates. Backtracked states and hyper-translocated states are evident and vary according to the substitution that is mapped. Wild-type RNAP, R780K, R780Q, G782A, G778A and A779V respond strongly and appropriately to addition of CTP, as expected. None of these RNAPs shows a strong defect in transcription (more than ∼2-fold down; Fig. 6). Strongly defective RNAPs K781A and G778A/G782A, however, appear to abolish CTP stabilization of the post-translocated TEC, consistent with the *K*_D_ measurements shown in Table 1. Very similarly to highly defective K781A, K781G/D/Q/R/E substitutions did not demonstrate CTP stabilization of the post-translocated TEC (data not shown).

For mapping RNAP excursions downstream (Fig. 10B and D), toward the post- and hyper-translocated states, exonuclease III attacks the upstream TEC boundary on the template DNA strand (3′→5′) (see Fig. 9). An RNA 3′-H_2_ chain terminator is present to block ATP (the next accurately templated NTP) incorporation. Mapping the upstream boundary, ATP strongly stabilizes the post-translocated register and suppresses hyper-translocation. Little effect is observed on pre-translocated or backtracked states. Once again, wild-type R780K/Q, G782A, G778A and A779V, with minor transcriptional defects, are strongly stabilized by ATP in the post-translocated register, as expected. Strongly defective, K781A and double substitution G778A/G782A do not appear to be stabilized by ATP binding, consistent with the *K*_D_ measurements shown in Table 1. Other K781 substitutions were not stabilized in the post-translocated register by ATP.

**Figure 11.**
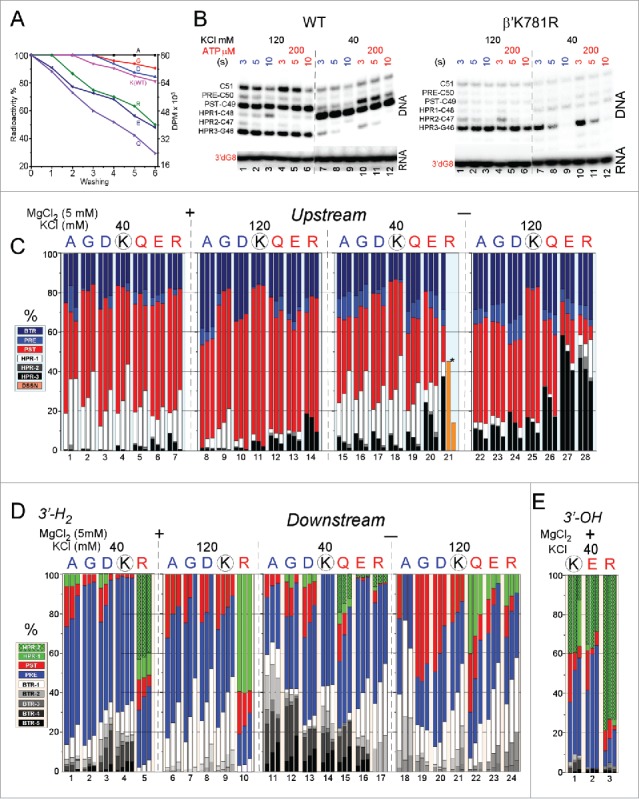
Some TECs containing K781 substitutions show sensitivity to termination, particularly when assembled in the absence of MgCl_2_. (A) RNAP TECs with K781Q/R/E substitutions are sensitive to washing with 1 M KCl buffer. (Colors in A do not relate to other colors in the figure.) (B) Mapping the upstream TEC boundary for TECs formed in the absence of MgCl_2_. K781R TECs formed in the absence of MgCl_2_ are unstable and terminate in the HPR3→HPR4 transition, particularly at 40 mM KCl. (C) Quantification of exonuclease III mapping of the upstream TEC border under various conditions. Orange bars indicate that for these samples TEC termination makes quantification difficult. (D) Quantification of exonuclease III mapping of the downstream TEC border under various conditions. Wild-type RNAP samples with four bars represent 3, 5, 10 and 20 seconds digestion with exonuclease III. Samples with three bars represent 3, 5 and 10 seconds digestion with exonuclease III. (E) Quantification of exonuclease III mapping of the downstream TEC border with a 3′-OH instead of a 3′-H_2_. In exonuclease III mapping, none of the K781 substitutions appears to respond to addition of accurately loaded NTPs (i.e., Fig. 10C and D), so these data are not shown in this figure.

Some of the K781 substitutions appeared to be particularly unstable for in vitro TEC assembly, so K781A/G/D/R/E/Q were compared to wild-type K781 RNAP for their ability to form TECs stable or unstable to repeated washing (Fig. 11A). TECs immobilized on beads were washed 0–6X with buffer containing 1 M KCl. K781A/G/D appeared to form more stable TECs than wild-type RNAP. By contrast, K781R/E/Q TECs appeared to be less stable to salt washing than wild-type TECs. As we show below, the most unstable TECs (K781R/E/Q) tend to hyper-translocate and to terminate transcription during exonuclease III mapping experiments. The more stable TECs (K781A/G/D) tend rather to backtrack and resist hyper-translocation and termination during exonuclease III mapping. Interestingly, sensitivity to washing also correlates with lower transcriptional activities (Fig. 6B and C). We concluded from this comparison that K781 substitutions fall into two broad classes with different properties attributable to a tendency to slide either upstream or downstream relative to wild-type RNAP. Furthermore, K781 substitutions give insight into the complexity of the N-terminal bridge helix hinge. Notably, the hinge appears to be governed by (1) flexibility; (2) R780 contacts to the fork; (3) two glycine bend points; and (4) alternating contacts of K781 and/or K781 substitutions that decrease or increase hinge flexibility. A more flexible hinge (i.e., K781G/A/D) supports backtracking. A less flexible hinge or one that might be expected to stick in one mode or another (i.e., K781Q/R/E) supports hyper-translocation and termination.

To analyze K781 substitutions that have low transcriptional activity and poor response to NTP loading, a more complex exonuclease III experiment was required. Using wild-type RNAP, we found that TECs assembled in the absence of Mg^2+^ gave similar results in exonuclease III experiments to TECs assembled in the presence of 5 mM Mg^2+^ (our standard procedure), but TECs assembled in the absence of Mg^2+^ had slightly altered but useful properties (Fig. 10A and B). Notably, compared to wild-type RNAP, sliding of TECs assembled in the absence of Mg^2+^ was magnified in K781G/A/D/Q/R/E. Mg^2+^ is required for exonuclease III digestion of DNA, so, when withheld during TEC assembly, Mg^2+^ was added during the 3–20 seconds incubation with exonuclease III.

In Fig. 10A and B, the two TEC assembly protocols are compared for wild-type RNAP. As noted above, this experiment demonstrates RNAP furthest excursions from a hyper-translocated toward a backtracked state. Because wild-type RNAP is analyzed, CTP effects are shown, and translocation registers can be confirmed. CTP stabilizes the post-translocated state, reduces the pre-translocated state and can reduce backtracking by holding the TEC in the post-translocated register. Additionally, experiments were done at 40 and 120 mM KCl (closer to physiological salt). We find that, at 120 mM KCl, TEC sliding is reduced, and the post-translocation register is stabilized with or without addition of NTPs. Furthermore, 120 mM KCl is expected to suppress backtracking that requires dissociation of the RNA 3′ end from the DNA template and is therefore favored at 40 mM KCl. When TECs are assembled in the absence of Mg^2+^ and when exonuclease III attacks the downstream TEC border, there is increased tendency for TECs to slide upstream.

The more complex exonuclease III mapping protocol provides insight into K781 substitutions (Fig. 11). For simplicity, for K781 substitutions, results in the presence of NTPs are not shown, because these results are in every case indistinguishable from results obtained in the absence of NTPs (i.e., as shown in Fig. 10C and D for K781A). Fig. 11C shows exonuclease III attack on the upstream TEC border, and RNAP excursions from the pre-translocated to the post-translocated and hyper-translocated positions. In summary, assembly of TECs in the absence of Mg^2+^ causes the TECs to slide further downstream and to hyper-translocate, particularly for K781Q/E/R. The observed decrease in HPR-3 signal with time of exonuclease III digestion indicates transcription termination, as TECs transition from HPR-3→HPR-4. Because the HPR-4 and HPR-4+ bands are not observed, termination appears to occur in the HPR-3→HPR-4 transition. In the case of K781R (40 mM KCl), termination is so evident (Fig. 11C, orange bars; Fig. 11B, right panel (K781R)), most TECs terminate, and, without assuming consistent recovery of samples, it is difficult to estimate what fraction of total TECs remain. K781A/G/D appear to resist hyper-translocation. K781Q/R/K appear to hyper-translocate more readily than wild-type RNAP. In this experimental context, K781R shows the greatest tendency to terminate transcription, and termination appears to occur in the transition HPR-3→HPR-4. 120 mM KCl appears to stabilize the post-translocated TEC compared to 40 mM KCl, particularly for wild-type RNAP. 120 mM KCl also slows termination in the HPR-3→HPR-4 transition. Because experiments were also done with addition of ATP (not shown), all mapping was done with a 3′-H_2_ RNA chain terminator.

When mapping with exonuclease III attack on the downstream TEC border, RNAPs furthest excursions upstream are tracked, so transitions are from post-translocated to pre-translocated to backtracked states (Fig. 11D) To summarize the experiment, small side chain substitutions K781A/G/D may tend to backtrack, particularly at 40 mM KCl. As expected, 120 mM KCl, which suppresses RNA/DNA strand separation, suppresses backtracking. K781Q/E/R tend to hyper-translocate and, therefore, not to backtrack, consistent with the data in Fig. 11C. Interestingly and unexpectedly, RNAs with a natural 3′-OH tend to hyper-translocate (to HPR-2) relative to RNAs with a 3′-H_2_ chain terminator (Fig. 11E). Hyper-translocation is particularly evident for K781R with a natural 3′-OH RNA.

We conclude that K781A/G/D and K781Q/R/E fall broadly into two mutant classes. K781A/G/D tend toward backtracking. K781Q/R/E tend toward hyper-translocation and, under some conditions, termination, as also observed in Fig. 11A with repeated salt washes of TECs. These data are consistent with a bridge helix hinge that governs translocation with multiple bend points, complex dynamics and tuning through the contacts of K781. Unexpectedly, when Mg^2+^is added late to reactions, tens of seconds are required to reestablish RNAP grip to RNA–DNA, indicating that bound Mg^2+^ is buried deeply in TEC positions that are largely shielded from solvent and, therefore, difficult to exchange.

For comparison, the transcriptional properties of bridge helix residues β′ H777A, K789A, G794P and Y795A were also analyzed (data not shown). H777A, just N-terminal to the 778-GARKGL-783 hinge, is slow in elongation (∼3-fold down compared to wild-type RNAP). K789A is very similar to wild type for transcriptional activity and NTP loading. Although proline substitution is generally disruptive of helices, G794P is only about 2-fold down for transcriptional activity compared to wild-type RNAP and is only somewhat defective for NTP loading. Y795A shows no obvious defect in transcription or stable NTP loading. Substitutions in the more C-terminal bridge helix 794-GY-795 hinge, therefore, are very similar to wild type, showing the primary importance of the N-terminal 778-GARKGL-783 hinge compared to the C-terminal 794-GY-795 hinge for RNAP elongation.

## Discussion

### Hinge action accompanying RNAP translocation

Using molecular dynamics simulation and biochemical approaches, we support the idea that hinges on the bridge helix and the trigger loop support a broad conformational change associated with forward translocation. In this and previous work, we indicate that the bridge helix N-terminal hinge GARKGL and two GXP hinges in the trigger loop helices GEPG and GLP are important.^19,25^ We show a molecular dynamics simulation (Figs. 1–4; Movies 1–3) that strongly supports dynamics of the trigger loop GEPG hinge and bending of the more C-terminal GLP hinge in translocation. Mutational analyses support the view obtained from dynamics.

Mutagenesis of the bridge helix GARKGL indicates that this is a complex, homeostatic hinge, with two glycine bend points. Either one of the glycines supports much of the necessary hinge dynamics, but mutation of both is much more strongly defective in transcription. The arginine mounted within the hinge forms a “ball in socket” arrangement in the β fork, coupling bridge helix hinge and fork dynamics. Substitution of this arginine generated a fast mutant R780K, a reasonably neutral mutation R780Q and a very strongly defective mutant R780A. The methyl side chain of R780A is not charged and is too short to penetrate the β fork. In the adjacent residue, K781 substitutions have varied and interesting properties. K781E/R/Q substitutions tend to hyper-translocate and terminate and induce instability of TECs to washing in 1 M KCl. For bacterial RNAP, the lysine side chain flexibility appears important to support hinge homeostasis. We posit that K781 makes alternate contacts to β D546 on the fork and to β′ D785 on the bridge helix. Because arginine is much less flexible than lysine, K781R appears to become stuck making just one contact or the other, disrupting the hinge homeostasis, i.e., by forcing the hinge too far in one or the other direction. The analysis here supports the idea of a complex homeostatic hinge with primary G778 and G782 bend points. The complex hinge is coupled to the fork through R780 and tuned by alternating ionic contacts of β′ K781 to β D546 and β′ D785. Because the trigger loop N-terminal helix is closely coupled to the bridge helix, one major function of the GARKGL N-terminal hinge appears to be to maintain bridge helix-trigger loop coupling during catalysis and translocation. Simulations indicate that the trigger loop may overtighten to support chemistry and/or bond completion (Figs. 1 and 2; Movie 1) and that trigger loop overtightening may help support forward translocation.

### A conformational model for translocation

All atom molecular dynamics simulation posits a surprisingly simple conformational model for RNAP translocation. First, the downstream i+2 base pair appears to break. Then, the trigger loop GLP C-terminal hinge bends, overtightening the trigger loop against the i+1 (active site) base pair. The bridge helix hinge GARKGL and the trigger loop hinge GEPG help to compensate for the greater GLP hinge bending. Forward translocation force generated in part by trigger loop overtightening then appears to open the furthest upstream i−8 RNA–DNA bp, setting up the possibility of forward TEC sliding. Simultaneously, the RNAP cleft and clamp close, indicating that RNAP may undergo a large conformational change during translocation. The RNA exit channel is also remodeled during simulation so that the most N-terminal β′ Zn finger and the β flap tip helix engage strongly with the exiting RNA (Fig. 4; Movie 2). Coupling the flap domain to RNA exit indicates that translocation dynamics communicate from the active site to the flap tip during elongation and also that the active site, β DPBB, flap domain (sandwich barrel hybrid motif) and flap tip helix are coupled to RNA threading through the exit channel (see Fig. 4; Movie 2).

### Translocation stickiness and RNAP grip to RNA–DNA

In this paper, we posit a conformational model for RNAP translocation. Such a model is consistent with a fairly stiff translocation ratchet, as opposed to a rapidly oscillating ratchet. Using variations in exonuclease III mapping procedures, we show effects of K^+^, Mg^2+^ and the RNA 3′-OH in regulating the sliding and grip of RNAP on DNA and RNA–DNA. Increased K^+^ suppresses backtracking and hyper-translocation. The effects of K^+^ on backtracking and hyper-translocation are posited to be primarily due to KCl inhibition of melting the RNA–DNA hybrid at the RNAP active site. Mg^2+^ appears to enhance the grip of RNAP on the RNA–DNA hybrid. When assembled in the absence of Mg^2+^, TECs are less stable. This is particularly true of K781E/R/Q substitutions, which tend to slide forward, hyper-translocate and, depending on conditions, terminate. Interestingly, termination due to hyper-translocation appears to occur at the HPR3→HPR4 transition, because HPR4 and further hyper-translocated TECs are not observed in exonuclease III mapping experiments, and HPR3 accumulates at 120 mM KCl but decays sharply from 3–20 seconds at 40 mM KCl, a condition that promotes TEC sliding.

Deeply buried Mg^2+^ may bridge ionic contacts, for instance, with aspartic acid residues, to DNA and RNA phosphates. We note that, when Mg^2+^ is added back to TECs, the Mg^2+^-mediated grip on DNA is not re-established immediately, as indicated by exonuclease III mapping experiments in which TECs can continue to slide even after Mg^2+^ re-addition (Fig. 10B, right panel; Fig. 11C). Effects of the RNA 3′-OH versus a 3′-H_2_ are also affected by some β′ GARKGL hinge substitutions (compare Fig. 11D and E). As judged by exonuclease III attack of the upstream TEC boundary, a 3′-OH RNA end tends to slide farther forward relative to a 3′-H_2_ end.

## Materials and methods

### All atom molecular dynamics simulations

Molecular dynamics simulations were done essentially as previously described.^19^ Tt RNAP PDB 2O5J with some missing residues from PDB 2O5I was used to construct the initial TEC structure with a closed trigger loop. ATP was substituted for AMPCPP in the RNAP active site. Otherwise, no changes were made in the structure. The TEC was simulated using CHARMM^28,29^ force fields and simulation data were analyzed using the MMTSB tool set. The simulations were done on the Texas Advanced Computing Center Stampede Supercomputer (XSEDE allocation: “Translocation by multi-subunit RNA polymerases” to ZFB and RIC (TG-MCB120005)). A closed TEC simulation of Ec RNAP cannot reasonably be done because no closed Ec RNAP TEC structure is available. Ec RNAP has the SI3 insertion in the more C-terminal trigger helix, so a reliable model for a closed Ec RNAP TEC is difficult to construct from Tt RNAP structures.

It is possible that the simulation shown in Figs. 1–4 and Movies 1–3 is an exaggerated representation of the translocation conformational change that RNAP undergoes during most bond addition cycles. Because phosphodiester bond formation does not occur in the simulation, equilibration may not be achieved. Also, the clamp can overtighten in the simulation because elongation factors that maintain but also might limit clamp closure (i.e., NusA, NusG) are absent. We have not generated the product complex (incorporated 3′ AMP.PPi) and relaxed the trigger loop at the end point of the current simulation to see whether the translocation step would then be completed, although we consider this a reasonable outcome and attractive model for bond completion.

### RNAP mutagenesis

RNAP mutagenesis, RNAP production and purification were done essentially as previously described.^26,27^

### In vitro transcription

RNA was 5′-end-labeled with ^32^P. Elongation was with 200 μM each NTP.^26,27^ Elongation rate (Fig. 6) was estimated compared to wild-type Ec RNAP. The number reported represents the ratio of the time for a mutant RNAP to reach an elongation state distribution versus the time required for wild-type RNAP to reach the same elongation distribution (expressed as approximate fraction of wild-type RNAP elongation rate).

### NTP affinity for RNAP

Transcription rate was measured at multiple NTP concentrations. A graph of rate versus NTP concentration was fit to the equation rate = *k*_pol_[NTP]/(*K*_D_+[NTP]). Error is standard error. Data were fit using the program Origin.

### Competition fidelity assay

The competition fidelity assay was run as previously described.^26,27^ Error is standard deviation of three measurements.

### Exonuclease III mapping

Mapping was done as previously described.^26,27^ Short RNAs are selected to maximize the effects of accurately loaded NTPs on translocation register. Briefly, TECs were assembled as described in Fig. 9. DNAs were 5′-end-labeled with ^32^P on the strand to be attacked with exonuclease III. The unlabeled DNA strand was blocked by incorporation of a 3′-thio-NMP for digestion by exonuclease III, so only the ^32^P-labeled DNA strand was a substrate for exonuclease III. The 3′ ends of probes (Fig. 9) were near to the RNAP boundaries so that digestion times could be short, in order to capture relevant translocation states. Controls with incoming NTP substrates (Fig. 10) indicate that the strategy is successful and that translocation states can be identified.

### Assignments of translocation states

Assignments of translocation states are made in the following way. The state most strongly stabilized by addition of the accurately loaded NTP (in the presence of a 3′-H_2_ chain terminator) is assigned as the post-translocated register, and other registers are assigned by comparison. This is an interpretation of the data with some potential limitations, and exonuclease III mapping data have been interpreted in slightly different ways by others. However, expressing the data in this manner makes it more intuitive for the reader to interpret. Digestions were done in excess exonuclease III in order to maximize exonuclease III rates. DNA templates were designed to maximize exonuclease III rates of attack on the RNAP boundary. For instance, exonuclease III prefers to hydrolyze CMP from the DNA 3′-end compared to other NMPs and DNA strands are designed for rapid digestion.

## Supplementary Material

1330179_Supplemental_Material.zip
