# Supplementary material for: Hinge action versus grip in translocation by RNA polymerase
Source: Transcription. 2017 Aug 30;9(1):1–16. doi: 10.1080/21541264.2017.1330179 (PMC5791816; doi:10.1080/21541264.2017.1330179)
Supplement: 1330179_Supplemental_Material.zip [file ktrn-09-01-1330179-s001.zip › Supplemental Movie Captions.docx]

**Movie 1.** Trigger loop dynamics and hinges. The template DNA strand is green. The RNA is white. The i+1 base pair and some trigger loop residues are colored for chemistry. The GLPG and GEP trigger loop hinges are indicated in stick representation. Mg is shown as magenta spheres.

**Movie 2.** Remodeling of the RNA exit channel. RNA is green. The most N-terminal β′ Zn finger is orange. The flap tip helix is gray. Some basic residues that engage the RNA are indicated.

**Movie 3.** Closing of the cleft. TDS is green. The ATP substrate (i+1) is shown. Some cleft residues are shown that form ion pairs as the cleft closes.
